# Supplementary material for: Disease-causing MFN2 mutants impair mitochondrial fission dynamics by distinct DRP1 dysregulation
Source: Cell Death Dis. 2026 Jun 5;17(1):707. doi: 10.1038/s41419-026-08838-3 (PMC13458151; doi:10.1038/s41419-026-08838-3)
Supplement: Supplementary file 1 [file 41419_2026_8838_MOESM1_ESM.pdf]

Supplementary figure legends

Figure S1. Methodology for quantification of mitochondrial fusion events. (**A**) Schematic representation of the mitochondrial fusion assay. Cells expressing matrix-targeted photoactivatable GFP (mtPA-GFP) and matrix-targeted DsRed (mtDsRed) were used to visualize mitochondrial fusion dynamics. ROIs of 5x5 µm were first photoactivated to locally induce mtPA-GFP fluorescence (1), followed by apposition of photoactivated and non-photoactivated mitochondria (2), and finally fusion, detected as matrix content mixing (3). (**B**) Representative time-lapse confocal images showing a mitochondrial fusion event. Mitochondria labelled with mtDsRed (red) and mtPA-GFP (green) were imaged at the indicated time points before (time 0s) and after photoactivation. The **green arrow** marks the donor mitochondrion (photoactivated mtPA-GFP), and the **orange arrow** marks the acceptor mitochondrion (non-photoactivated mtDsRed). The **white arrow** indicates the apposition site where mitochondria come into close contact before fusion, and the magenta arrow marks the fusion site, where matrix mixing occurs, and mtPA-GFP fluorescence spreads into the acceptor mitochondrion. Scale bar: 10 µm. (**C**) Line-scan fluorescence intensity profiles of mtPA-GFP (green) and mtDsRed (red) along the mitochondrial axis at the indicated time points. The progressive convergence of the two fluorescence intensity peaks demonstrates the mixing of matrix contents, confirming the occurrence of mitochondrial fusion.

Figure S2. L248H patient cells show increased interconnectivity, and CMT2A-causing mutants cannot rescue the mitochondrial fusion frequency and provoke mild defects in morphology upon acute overexpression in Mfn2-/- MEFs. (A) Fluorescence decay post photoactivation in the photoactivated ROI (white dotted squares, Figure 2A). Quantification after ≈ 5 and 70 s after mt-PAGFP photoactivation in cells analysed in Figure 2A (bar plot). (**B**) Mfn2-/- MEFs acutely co-expressing mtDsRed, mtPA-GFP, and WT or mutant MFN2. Representative images 20 s post-photoactivation of 5x5 µm ROIs (left). Insets displaying 1 or 2 ROIs highlighting the extent of mtPA-GFP diffusion and mitochondrial morphology (right). (**C**) Mitochondrial morphology classification and quantification using mtDsRed images in ≥ 30 cells. (**D**) Fusion events frequency (Mfn2-KO + E.v. = 19 cells, Mfn2-KO + MFN2 = 17 cells, Mfn2-KO + MFN2-L248H = 17 cells, Mfn2-KO + MFN2-M376V = 22 cells). (**E**) Representative Western blot from whole protein extracts of MEFs Mfn2-KO cells acutely

expressing MFN2 WT or mutants. Data are mean ± SEM of ≥ 3 independent experiments. **P< 0.01*,* ****P< 0.0001 *vs Control 1 or* MFN2.

Figure S3. MEFs stable-expressing CMT2A-causing mutants. (**A**) Representative Western blot from whole protein extracts of MEFs clones stably expressing WT or mutant MFN2. Clones highlighted in red were selected for the following experiments. (**B**) Selected clones were incubated for 20 min with TMRE (10 nM). Representative images and insets depicting mitochondrial morphology (dashed squares).

Figure S4. Cells expressing MFN2-L248H show a bead-on-string mitochondrial phenotype. (**A**) Representative images of Control or CMT2A-derived fibroblasts expressing mtDsRed showing mitochondrial matrix constriction (White arrows) (**B**) Number of mitochondrial matrix constrictions per mitochondrial length (μm) in human fibroblasts and (**C**) stable MEFs.

Figure S5. EYFP-DRP1 image pre-processing. (**A**) MEFs stable-expressing MFN2 or mutants, co-transfected with mt-BFP and EYFP-DRP1, were imaged every 3 s for 7 min upon 405 and 514 nm laser excitation. The threshold was defined according to the integrated density average of 5 high-intensity EYFP-DRP1 puncta (bottom, white circles). (**B**) Mitochondrial fission example where EYFP-DRP1 high-intensity puncta (right, green arrowheads) concur with mitochondrial constriction (left, histograms).

Figure S6. MFN2 overexpression decreases mtDNA levels in stable MEFs. Total DNA extracted from stable MEFs expressing WT MFN2 or mutants. (**A**) The mtDNA/nDNA ratio was assessed using qPCR targeting the mitochondrial ND5 gene and the nuclear β-actin gene. (**B**) Agarose gel of three different experiments (n1, n2, and n3) showing long-PCR products of mtDNA amplification using the following primers: **mtDNA 7.7 kb F** ACCTGAATTGGGGGCCAACC; **mtDNA 7.7 kb R** TGGCGAAGTGGGCTTTTGCT and **mtDNA 8.6 kb F** AGCAAAAGCCCACTTCGCCA; **mtDNA 8.6 kb R** GGTTGGCCCCCAATTCAGGT. Data are mean ± SEM of ≥ 3 independent experiments. ***P*<0.01, ***P*<0,001 *vs* E.v.

Figure S7. Seahorse OCR-based calculations. OCR-based calculations representing (**B**) respiration linked to ATP production, (**B**) Spare capacity, (**C**) Proton leak, and (**D**) Non-

mitochondrial respiration were plotted. (**E**) Coupling efficiency = (OCR ATP production)/(OCR Basal Respiration) x 100; each consisting of 12 technical replicates. (**F**) Glycolytic capacity, calculated as the difference between ECAR following the injection of 1 μm oligomycin and the basal ECAR. Data are mean ± SEM of ≥ 3 independent experiments. * $P<0.05$, ** $P<0.01$, **** $P<0.0001$ vs +MFN2.
