# Supplementary material for: Disease-causing MFN2 mutants impair mitochondrial fission dynamics by distinct DRP1 dysregulation
Source: Cell Death Dis. 2026 Jun 5;17(1):707. doi: 10.1038/s41419-026-08838-3 (PMC13458151; doi:10.1038/s41419-026-08838-3)
Supplement: Supplementary file 2 [file 41419_2026_8838_MOESM2_ESM.pdf]

Supplementary table 1. Antibodies

Target	Dilution	Source	Catalogue number
MFN2	1:2000	Abcam	ab50838
MFN1	1:2000	Abcam	126575
Flag	1:2000	Thermo Fisher	14-6681-37
OPA1	1:2000	BD Transduction Laboratories	612607
DRP1	1:2000	BD Transduction Laboratories	611113
pDRP1-Ser616	1:2000	Cell Signaling	3455
pDRP1-Ser637	1:2000	Cell Signaling	4867
MFF	1:2000	Proteintech	17090-1-AP
FIS1	1:2000	Proteintech	10956-1-AP
OXPHOS	1:5000	Abcam	Ab110413
TOMM20	1:2000	Cell Signaling	42406
GAPDH	1:5000	Proteintech	Cat #60004-1-Ig
Rabbit IgG HRP	1:5000	Jackson ImmunoResearch,	111-035-045
Mouse IgG HRP	1:5000	Jackson ImmunoResearch	115-035-062

Supplementary table 2. Plasmids

Name	Source	Backbone
MFN2	Donated by Dr. Gyorgy Hajnóczky	pECFP-C1
MFN2-L248H	This work	pECFP-C1
MFN2-M376V	This work	pECFP-C1
FUGW (E.v.)	Donated by Dr. Maria Estela Andrés	FUGW
MFN2-Flag	This work	FUGW
MFN2-L248H-Flag	This work	FUGW
MFN2-M376V-Flag	This work	FUGW
MFN2-R364W-Flag	This work	FUGW
EYFP-Drp1	Donated by Dr. Gyorgy Hajnóczky	pEYFP-C1
mtPA-GFP	Donated by Dr. Gyorgy Hajnóczky	pAcGFP1-Mito
mtDsRed2	Donated by Dr. Gyorgy Hajnóczky	pDsRed2-Mito
mtBFP	Addgene	pAcGFP1-Mito

Supplementary table 3. Primers and probes

Name	Source	Sequence
**Cloning primers**		
BamHI_Mfn2 FP	IDT, Integrated DNA Technologies	5'agcGGATCCATGTCCCTGCTCTTCTCTCG 3'
Mfn2_Flag_EcoRI RP	IDT, Integrated DNA Technologies	5'aatgGAATTCCTACTTATCGTCGTCATCCTTGTAATC ggcTCTGCTGGGCTGCAGGT 3'
**Quantification of mtDNA levels**		
mtND5 Forward	Thermo Fisher	5'ACCTAATTAAACACATCAACTTCCC 3'
mtND5 Reverse	Thermo Fisher	5'GACTCAGTGCCAGGTTGTAA 3'
β-actin Forward	Thermo Fisher	5'CTGCTCTTTCCCAGACGAGG 3'
β-actin Reverse	Thermo Fisher	5'AAGGCCACTTATCACCAGCC 3'
TaqMan mtND5	Thermo Fisher	5'-HEX -ACACCACCACATCAATCAAATTCTCCTTCA - BHQ_1 - 3'
TaqMan β-actin	Thermo Fisher	5'Probe-FAM-ATTGCCTTTCTGACTAGGTG-BHQ_1-3'
**Long Range PCR**		
mtDNA 7.7 kb Forward	Thermo Fisher	5'ACCTGAATTGGGGGCCAACC 3'
mtDNA 7.7 kb Reverse	Thermo Fisher	5'TGGCGAAGTGGGCTTTTGCT 3'
mtDNA 8.6 kb Forward	Thermo Fisher	5'AGCAAAAGCCCACTTCGCCA 3'
mtDNA 8.6 kb Reverse	Thermo Fisher	5'GGTTGGCCCCCAATTCAGGT 3'

Supplementary Table 4. Findings summary.

Cell model	Parameter	MFN2-L248H	MFN2-M376V
Patient´s cells	Ultrastructure<br>Morphology<br>Fusion<br>Fission	Empty/aberrant cristae<br>Hyper-elongated<br>Inhibited<br>Inhibited	Aberrant cristae<br>Fragmented<br>Inhibited<br>Unaltered
MEF acute OE	Morphology<br>Fusion	Hyper-elongated<br>Inhibited	Fragmented<br>Inhibited
MEF stable OE	Morphology<br>Fusion<br>Fission<br>mtΔψ<br>Basal OCR<br>Max. OCR<br>ECAR	Hyper-elongated<br>Inhibited<br>Inhibited<br>Augmented<br>Augmented<br>Augmented<br>Unaltered	Fragmented<br>Inhibited<br>Augmented<br>Augmented<br>Inhibited<br>Unaltered<br>Augmented

Comparative summary of the MFN2 mutants specific findings in CMT2A patient-derived cells and acute expression of CMT2A-causing mutants in MEF WT background. Changes are expressed compared to the control or WT MFN2 OE conditions. **mtΔψ**: Mitochondrial membrane potential; **OCR**: Oxygen consumption rate; **ECAR**: Extracellular acidification rate

Fig. S1

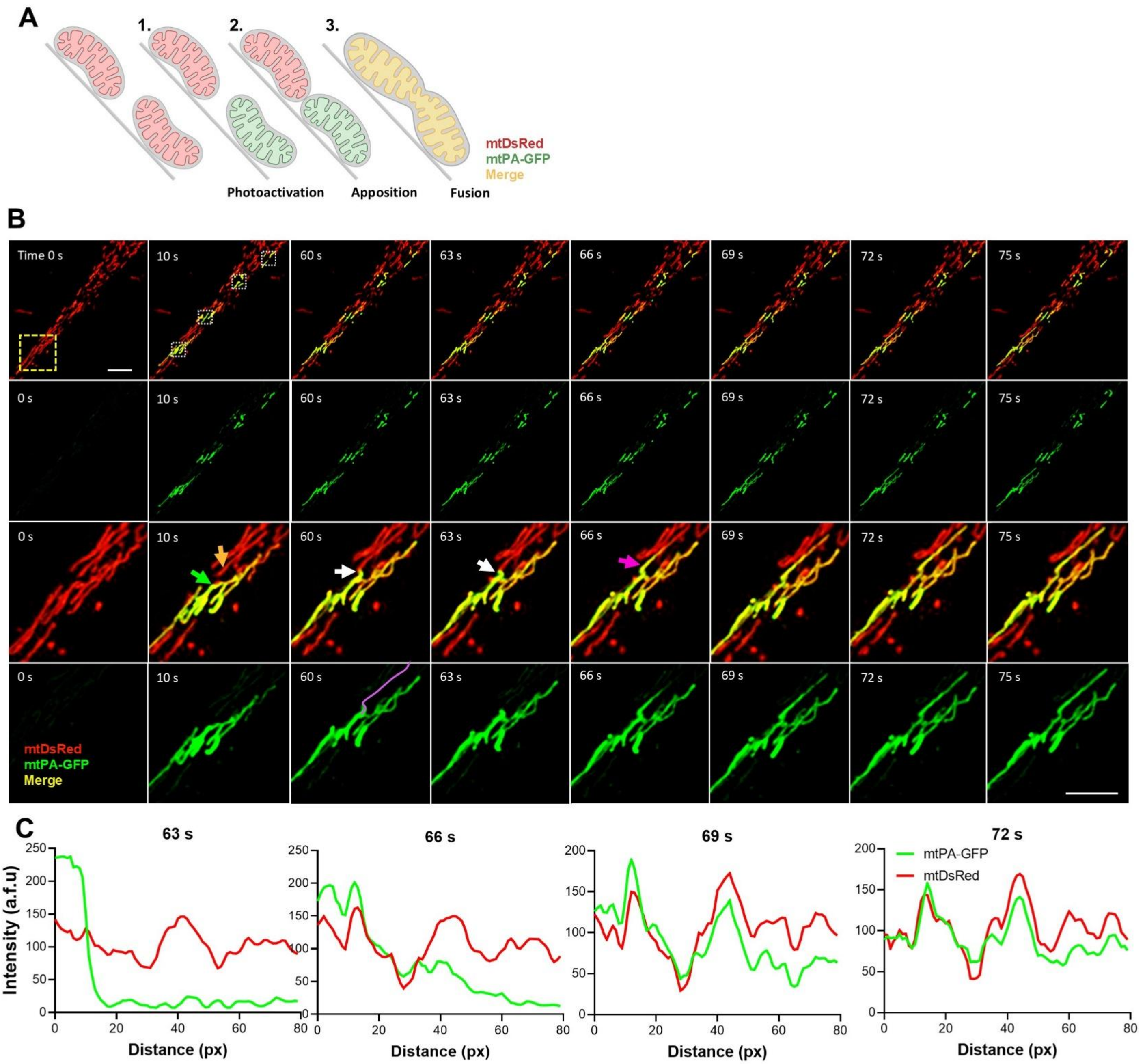
A
1.
2.
3.
mtDsRed
mtPA-GFP
Merge
Photoactivation
Apposition
Fusion
B
Time 0 s
10 s
60 s
63 s
66 s
69 s
72 s
75 s
mtDsRed
mtPA-GFP
Merge
C
63 s
66 s
69 s
72 s
Intensity (a.f.u)
Distance (px)
mtPA-GFP
mtDsRed

Fig. S2

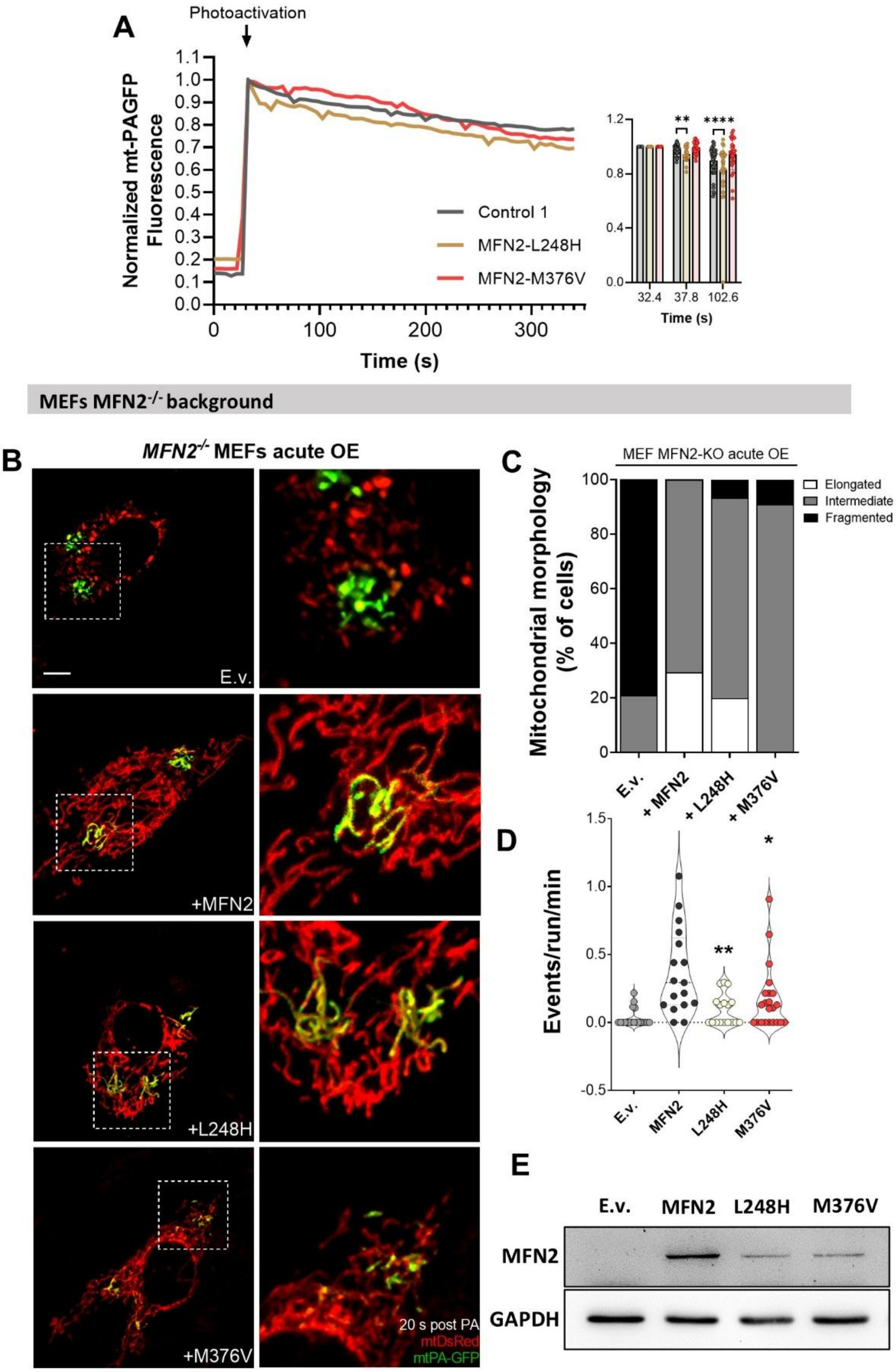

A
Photoactivation
Normalized mt-PAGFP Fluorescence
Time (s)
Control 1
MFN2-L248H
MFN2-M376V
**
****
32.4
37.8
102.6
Time (s)
MEFs MFN2-/- background
B
MFN2-/- MEFs acute OE
E.v.
+MFN2
+L248H
+M376V
20 s post PA
mtDsRed
mtPA-GFP
C
MEF MFN2-KO acute OE
Mitochondrial morphology (% of cells)
Elongated
Intermediate
Fragmented
E.v.
+ MFN2
+ L248H
+ M376V
D
Events/run/min
E.v.
MFN2
L248H
M376V
E
E.v.
MFN2
L248H
M376V
MFN2
GAPDH

Fig. S3

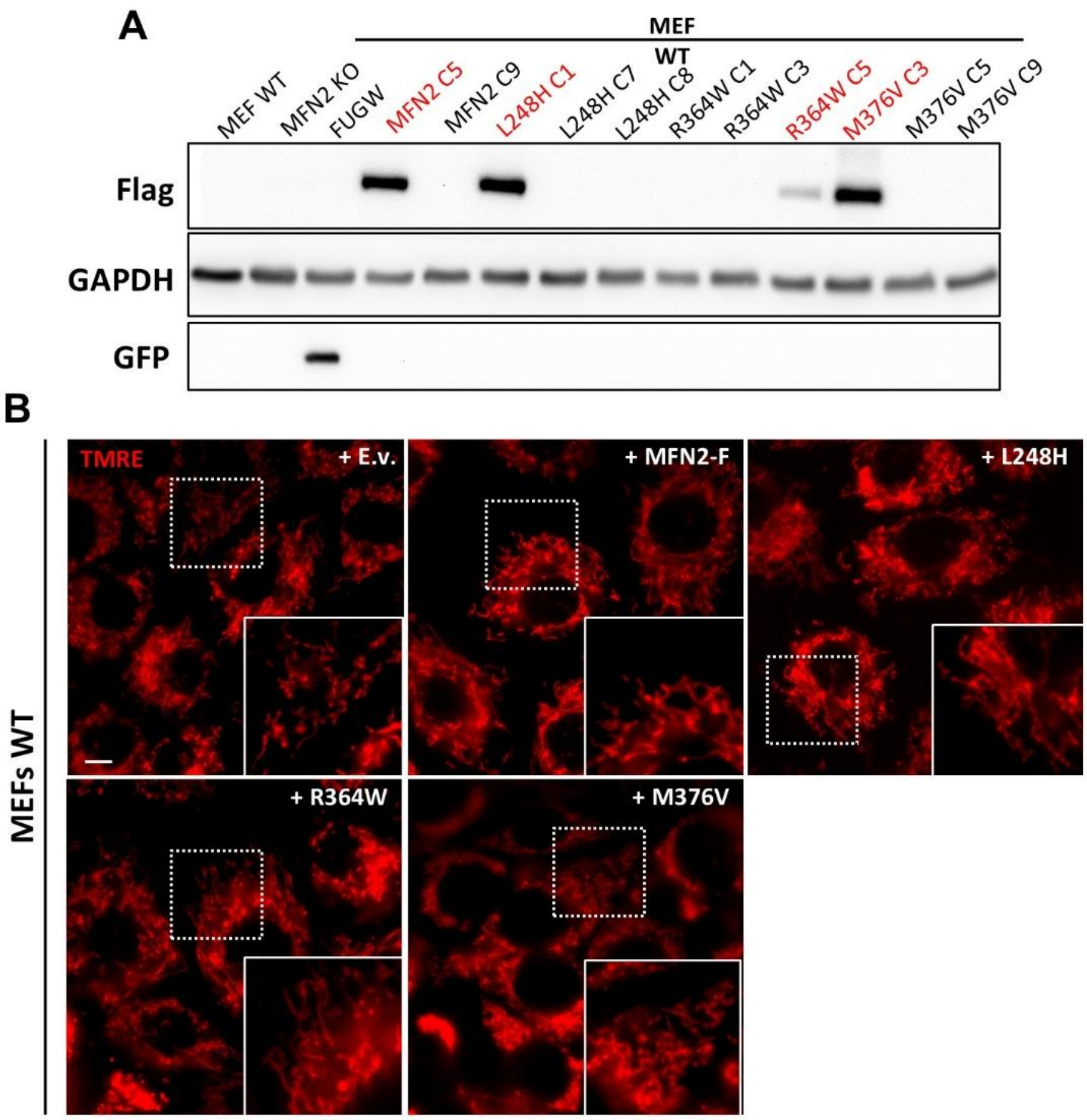
A
MEF
WT
MEF WT
MFN2 KO
FUGW
MFN2 C5
MFN2 C9
L248H C1
L248H C7
L248H C8
R364W C1
R364W C3
R364W C5
M376V C3
M376V C5
M376V C9
Flag
GAPDH
GFP
B
TMRE
+ E.v.
+ MFN2-F
+ L248H
+ R364W
+ M376V
MEFs WT

Fig. S4

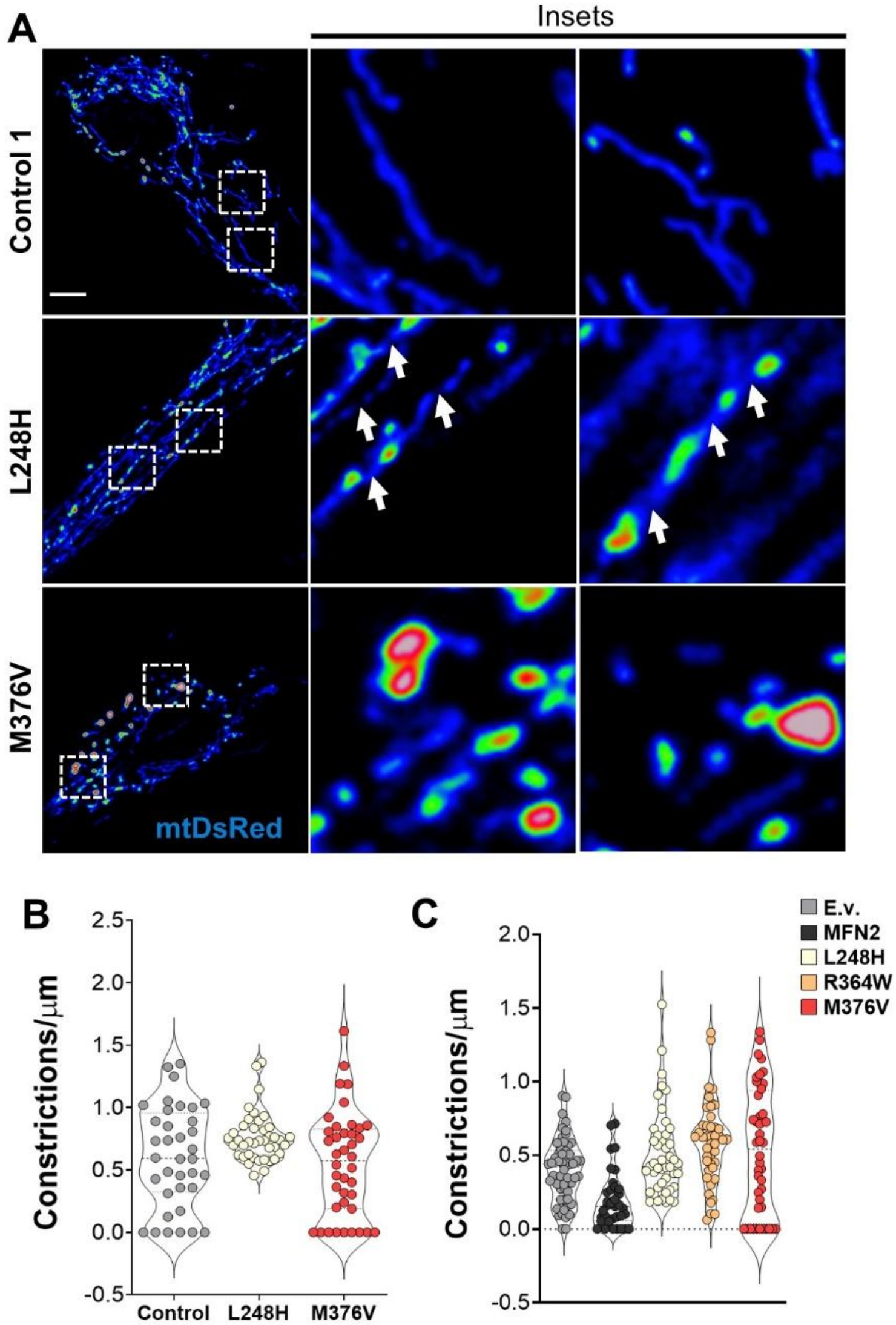
A
Insets
Control 1
L248H
M376V
mtDsRed
B
Constrictions/µm
2.5
2.0
1.5
1.0
0.5
0.0
-0.5
Control
L248H
M376V
C
Constrictions/µm
2.0
1.5
1.0
0.5
0.0
-0.5
E.v.
MFN2
L248H
R364W
M376V

Fig. S5

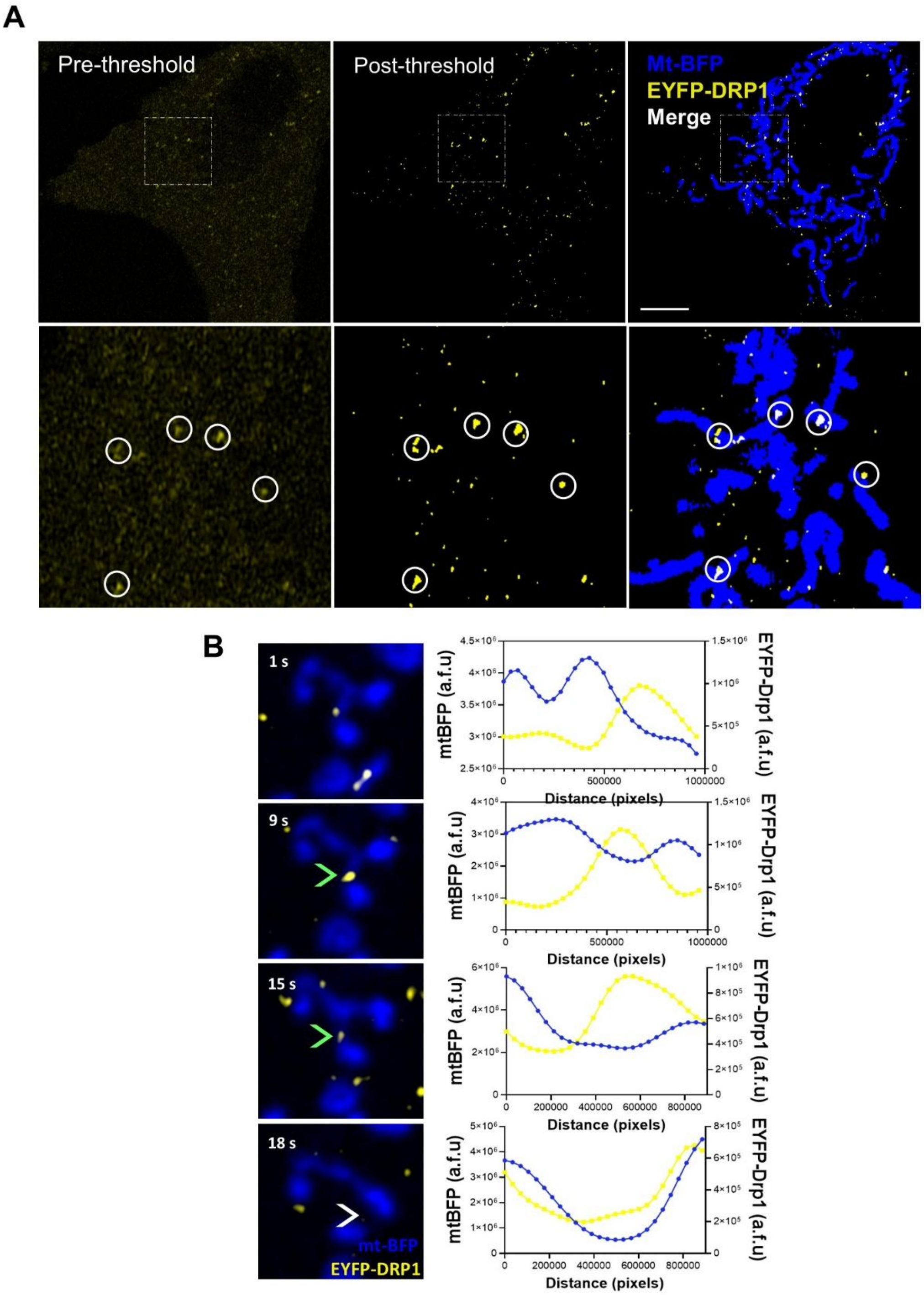

A
Pre-threshold
Post-threshold
Mt-BFP
EYFP-DRP1
Merge
B
1 s
9 s
15 s
18 s
mt-BFP
EYFP-DRP1
mtBFP (a.f.u)
EYFP-Drp1 (a.f.u)
Distance (pixels)

Fig. S6

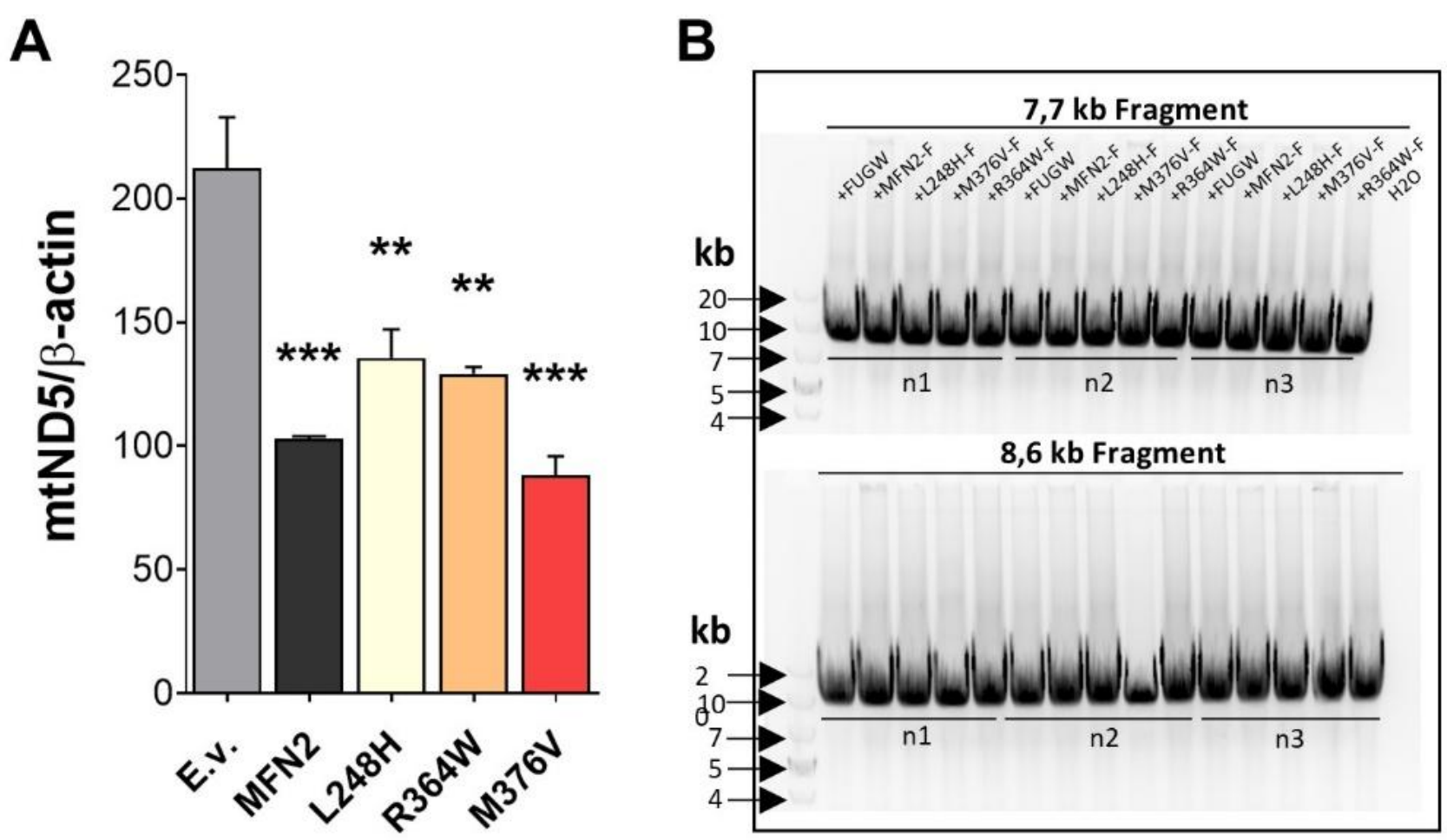
A
250
200
150
100
50
0
mtND5/β-actin
***
**
**
***
E.v.
MFN2
L248H
R364W
M376V
B
7,7 kb Fragment
+FUGW
+MFN2-F
+L248H-F
+M376V-F
+R364W-F
H2O
kb
20
10
7
5
4
n1
n2
n3
8,6 kb Fragment
kb
2
0
10
7
5
4
n1
n2
n3

Fig. S7

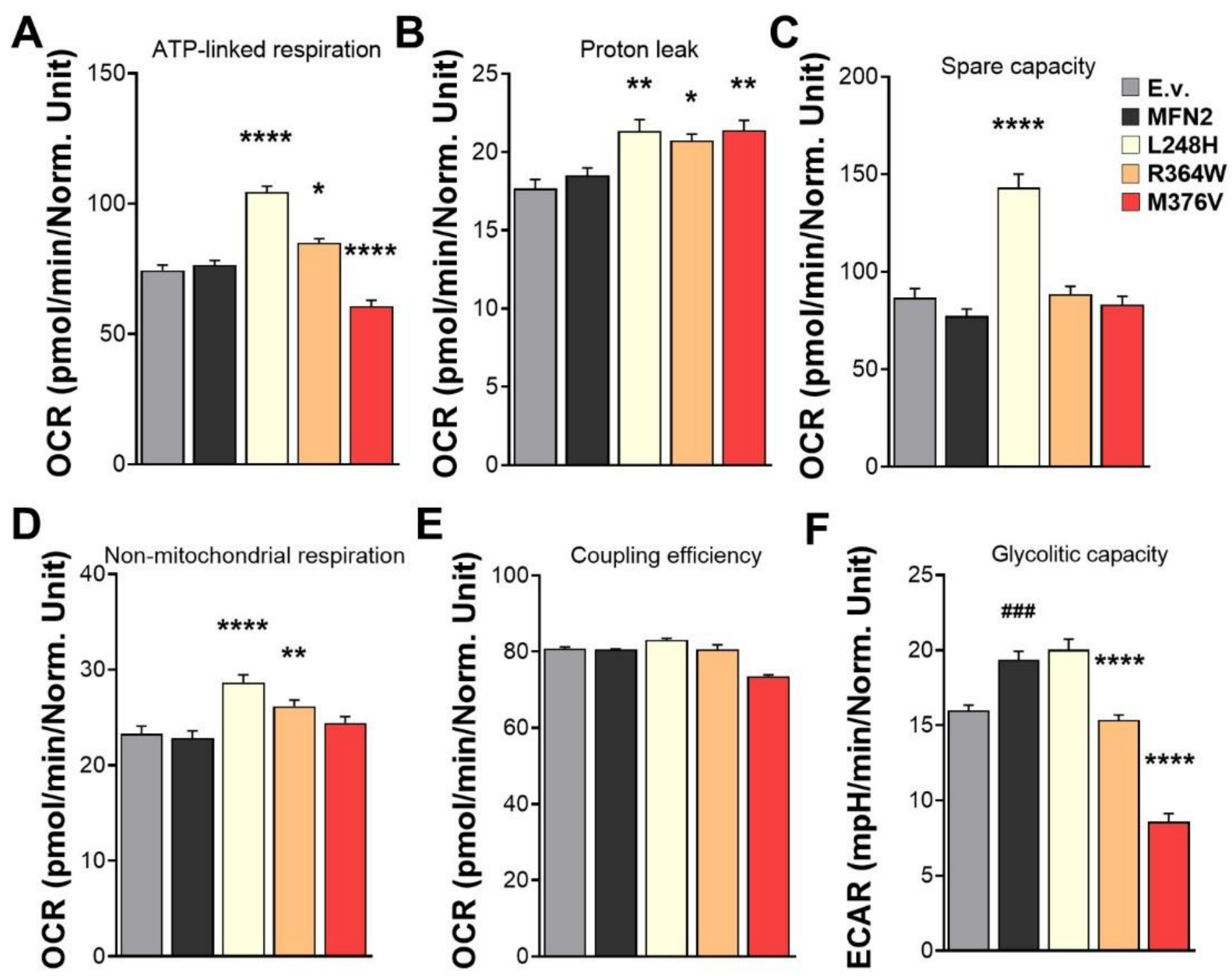
A
ATP-linked respiration
OCR (pmol/min/Norm. Unit)
150
100
50
0
****
*
****
B
Proton leak
OCR (pmol/min/Norm. Unit)
25
20
15
10
5
0
**
*
**
C
Spare capacity
OCR (pmol/min/Norm. Unit)
200
150
100
50
0
****
E.v.
MFN2
L248H
R364W
M376V
D
Non-mitochondrial respiration
OCR (pmol/min/Norm. Unit)
40
30
20
10
0
****
**
E
Coupling efficiency
OCR (pmol/min/Norm. Unit)
100
80
60
40
20
0
F
Glycolitic capacity
ECAR (mpH/min/Norm. Unit)
25
20
15
10
5
0
###
****
****
