# Supplementary material for: Disease-causing MFN2 mutants impair mitochondrial fission dynamics by distinct DRP1 dysregulation
Source: Cell Death Dis. 2026 Jun 5;17(1):707. doi: 10.1038/s41419-026-08838-3 (PMC13458151; doi:10.1038/s41419-026-08838-3)

Uncropped blots

Fig. 2D

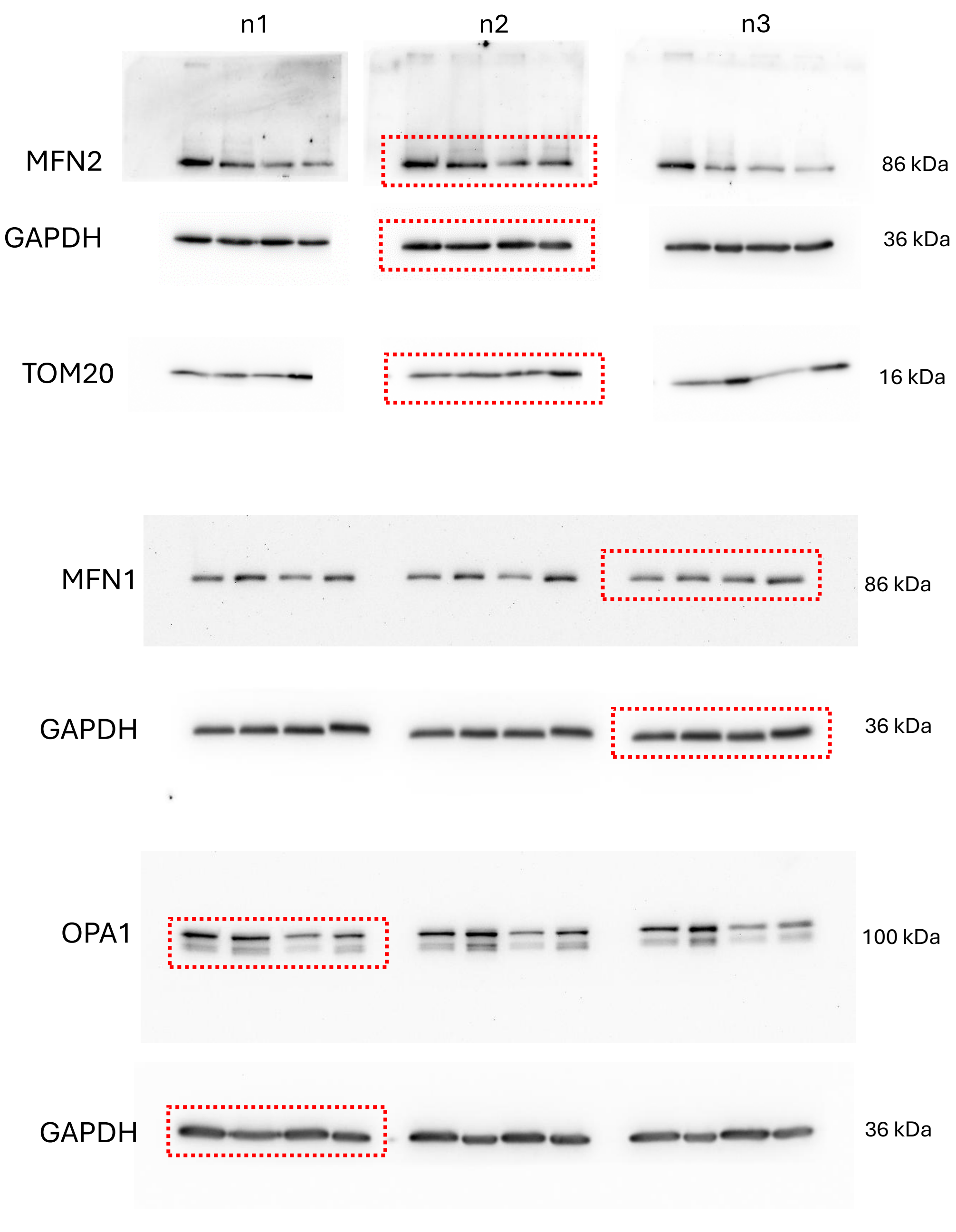

Fig. 4A

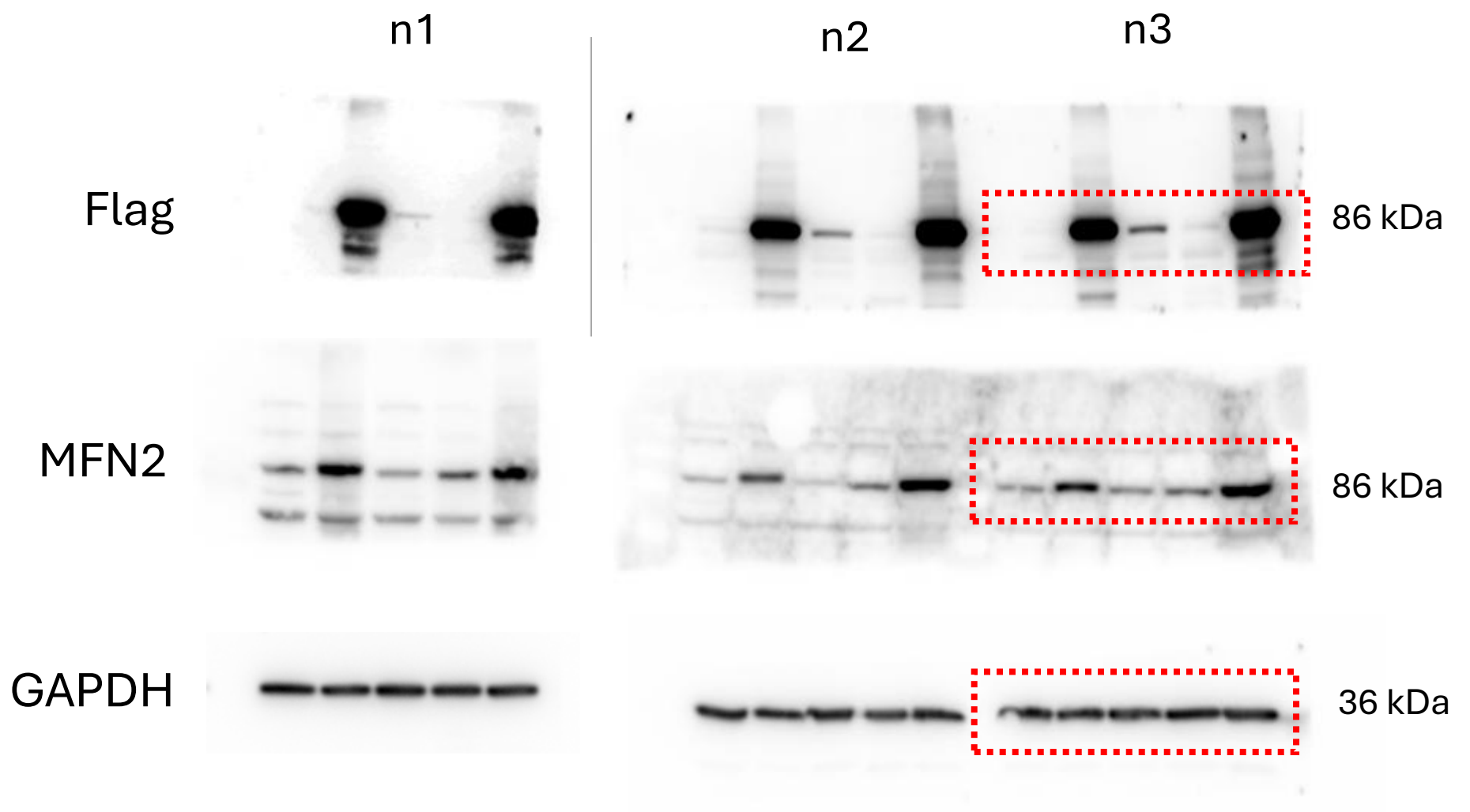

n1
n2
n3
Flag
MFN2
GAPDH
86 kDa
86 kDa
36 kDa

Fig. 4E

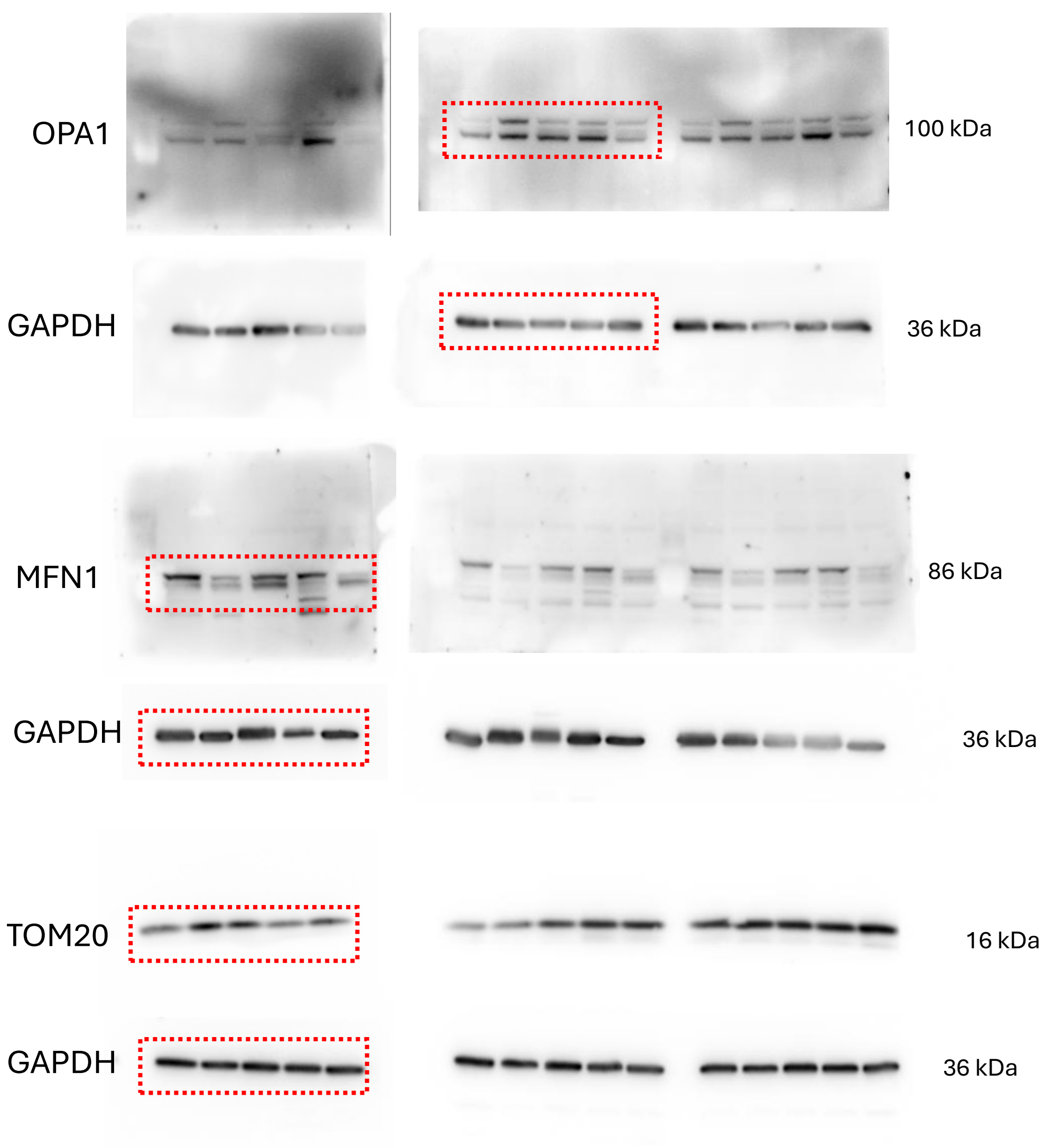

OPA1
GAPDH
MFN1
GAPDH
TOM20
GAPDH
100 kDa
36 kDa
86 kDa
36 kDa
16 kDa
36 kDa

Fig. 5E

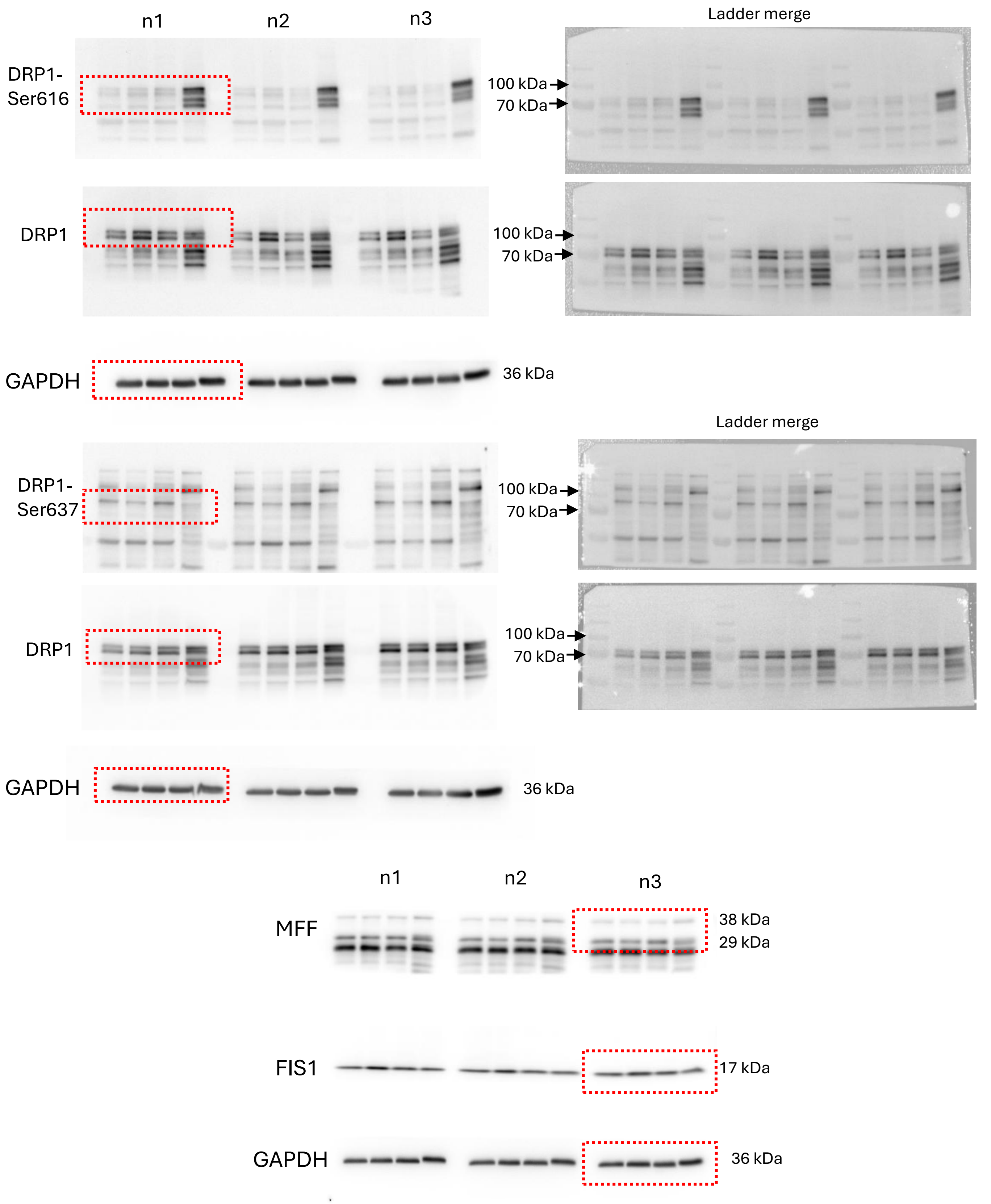

n1
n2
n3
Ladder merge
DRP1-
Ser616
100 kDa
70 kDa
DRP1
100 kDa
70 kDa
GAPDH
36 kDa
Ladder merge
DRP1-
Ser637
100 kDa
70 kDa
DRP1
100 kDa
70 kDa
GAPDH
36 kDa
n1
n2
n3
MFF
38 kDa
29 kDa
FIS1
17 kDa
GAPDH
36 kDa

Fig. 6E

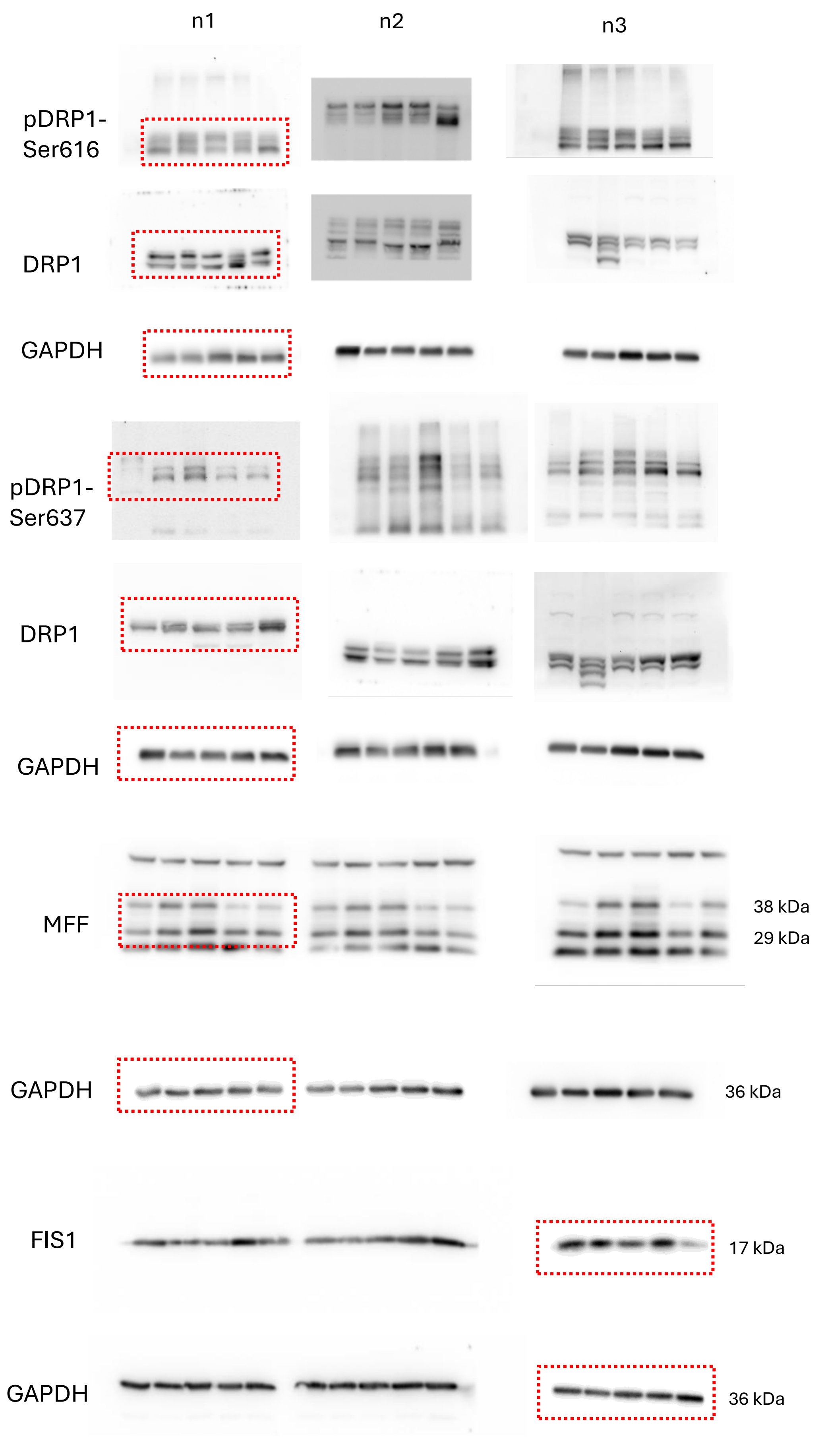

n1
n2
n3
pDRP1-Ser616
DRP1
GAPDH
pDRP1-Ser637
DRP1
GAPDH
MFF
38 kDa
29 kDa
GAPDH
36 kDa
FIS1
17 kDa
GAPDH
36 kDa

Fig. 7G

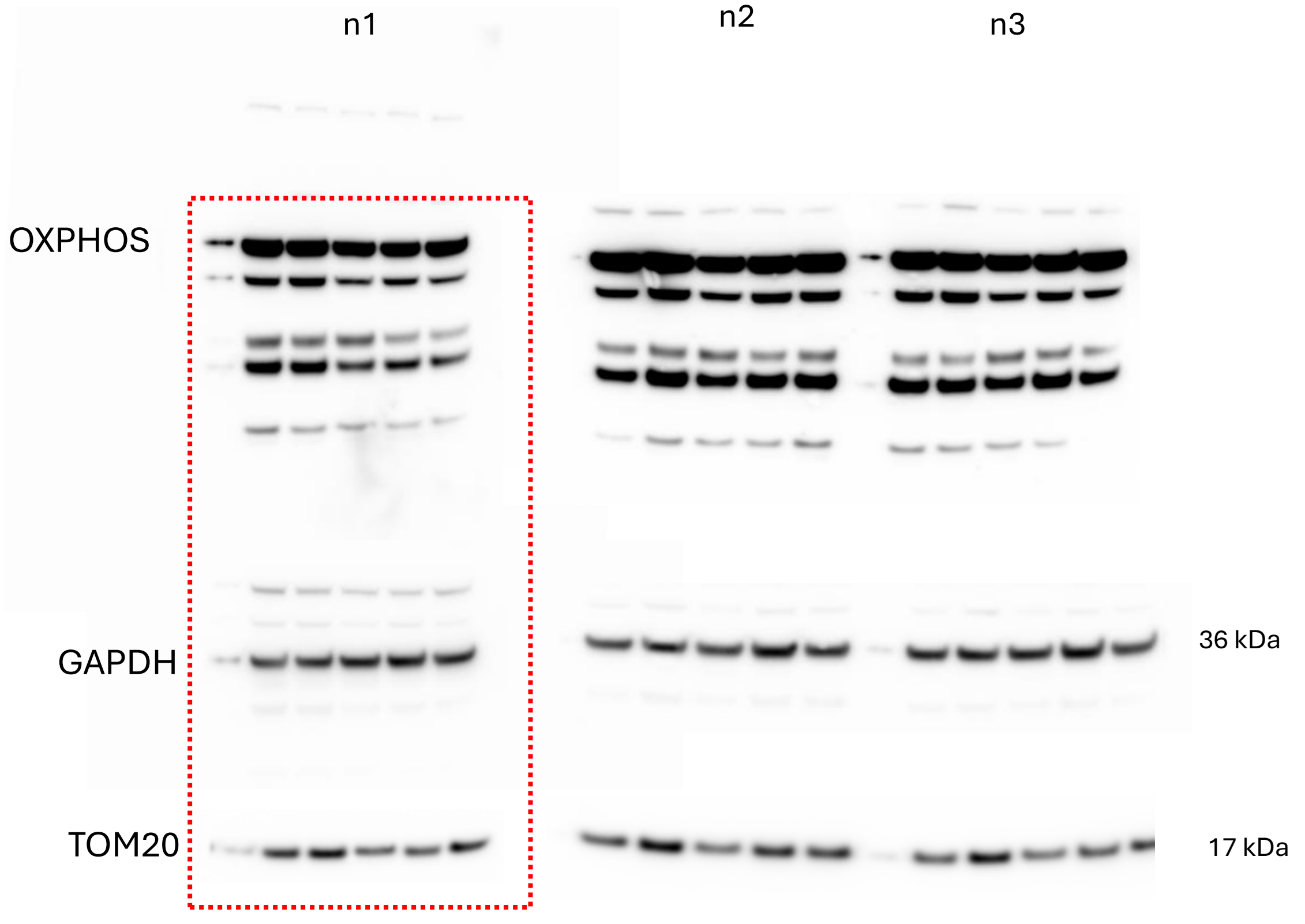

Supplement: Supplementary file 3 [file 41419_2026_8838_MOESM3_ESM.pdf]
